# Bidirectional roles of meningeal lymphatic vessels in brain metastases: friend and foe

**DOI:** 10.3389/fonc.2026.1793165

**Published:** 2026-05-13

**Authors:** Wei Shao, Erdi Zhu, Daoning Chen, Hua Lu

**Affiliations:** Department of Neurosurgery, Affiliated Hospital of Jiangnan University, Wuxi, China

**Keywords:** brain metastatic tumors, dual roles, immune regulation, meningeal lymphatic vessels, treatment strategies, tumor microenvironment

## Abstract

Brain metastases constitute the most prevalent intracranial malignancies. Despite recent therapeutic advances, patient prognoses remain poor, largely due to significant treatment resistance and a highly restrictive tumor microenvironment. The rediscovery of meningeal lymphatic vessels (MLVs) has provided new insights into how the brain interacts with the immune system. MLVs are responsible for cerebrospinal fluid drainage, waste clearance, and immune cell trafficking, and their dysfunction is linked to many neurological diseases. In brain metastases, MLVs play a dynamic, dual role based on the evolving tumor microenvironment. Under physiological conditions or in early stages, MLVs promote anti - tumor immunity by draining tumor antigens to cervical lymph nodes and supporting T-cell activation. However, as the tumor progresses, excessive tumor - derived factors like VEGF-C cause pathological MLV remodeling. This structural change is a critical switch: pathologically dilated MLVs facilitate tumor dissemination and their drainage dysfunction creates a local immunosuppressive niche, leading to immune evasion. This “friend and foe” character makes MLVs a potential therapeutic target. Enhancing MLV drainage may improve immunotherapy and drug delivery, while inhibiting tumor-driven lymphangiogenesis may help limit metastatic spread. In this review, we summarize current knowledge on MLV biology, their interactions with brain metastases, and discuss potential strategies and challenges for targeting MLVs in future therapies.

## 1 Introduction

Brain metastases are secondary tumors that originate from extracranial malignancies and colonize the brain parenchyma or meninges (1, 2). Epidemiological studies indicate that 20–40% of cancer patients will eventually develop brain metastases, most commonly from lung, breast, melanoma, renal cell, and colorectal primaries (3, 4). Compared with primary gliomas, these metastases exhibit greater invasiveness, higher recurrence rates, and more pronounced inter-lesional heterogeneity (5). The blood–brain barrier prevents most systemic therapies from reaching intracranial lesions, while the local tumor microenvironment is highly immunosuppressive (6). These limitations contribute to therapy resistance, and median survival for patients with brain metastases remains only a few months (7). These challenges underscore the urgent need for novel insights into the biology of brain metastases and new therapeutic approaches.

Recent advancements have fundamentally transformed the understanding of central nervous system (CNS) immunity through the discovery of functional meningeal lymphatic vessels (MLVs) that drain cerebrospinal fluid (CSF) and immune cells into the cervical lymph nodes (8, 9). Subsequent studies have revealed that MLVs contribute to waste clearance, cerebral metabolism, and immune surveillance (10). Dysfunction of MLVs has been linked to neurodegenerative and neuroinflammatory conditions, highlighting their importance for CNS homeostasis (11).

Increasing evidence also points to a role for MLVs in cancer within the CNS. In glioblastoma models, MLVs have been shown to influence antigen presentation and T-cell activation (12). Although our understanding of their specific mechanisms in brain metastases remains limited, accumulating data suggest that MLVs play a paradoxical role: they can promote anti-tumor immunity by draining antigens to support T-cell activation (13), while their tumor-driven remodeling can also provide a potential physical conduit to facilitate metastatic progression and extracranial dissemination (12). This dual ‘friend and foe’ nature positions MLVs as critical regulators of brain metastatic outcomes. Therapeutic manipulation of MLVs—whether to enhance immune responses (14) and drug delivery (15), or to block tumor-driven remodeling—is gaining attention as a novel strategy to overcome current treatment barriers. In this review, we summarize current understanding of MLV structure and function, examine their dual roles in brain metastases, and discuss emerging therapeutic opportunities and challenges for targeting MLVs in the context of metastatic disease.

## 2 Meningeal lymphatic vessels

### 2.1 Discovery of MLVs

**Figure 1:**
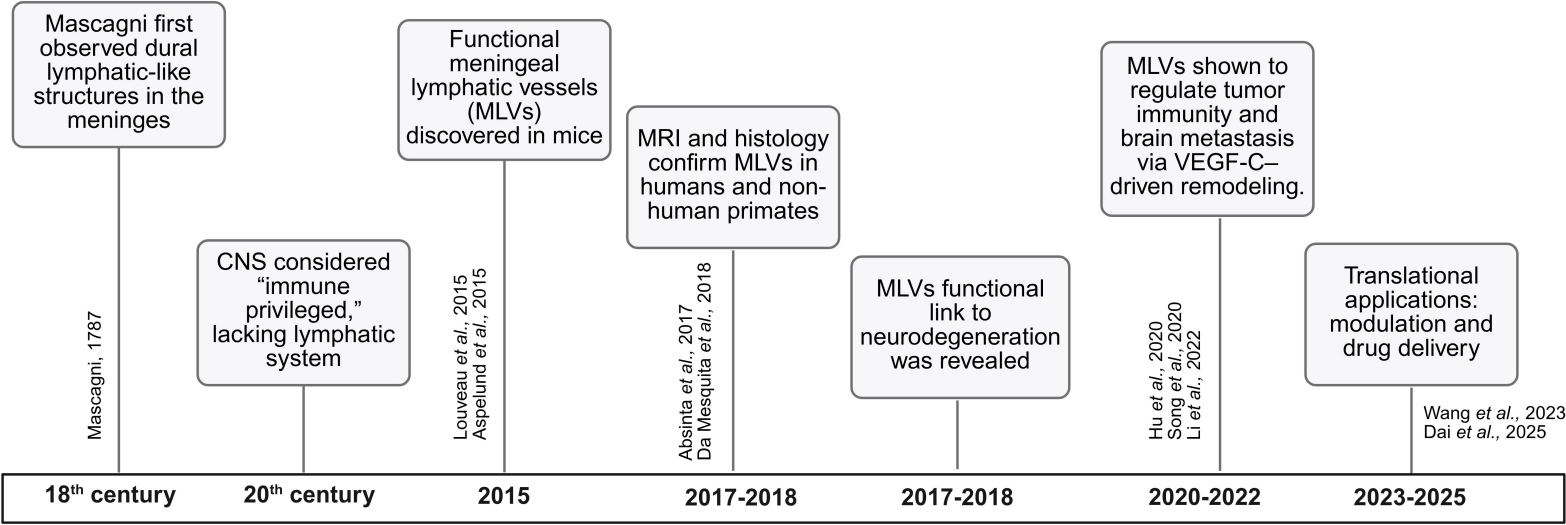
Timeline of key milestones in the discovery and functional characterization of meningeal lymphatic vessels (MLVs).

In the 18th century, the Italian anatomist Paolo Mascagni first observed dural structures resembling lymphatic vessels. Limited by the technology of the time, this pioneering observation failed to gain acceptance (16). Throughout the 20th century, the prevailing view was that the CNS was “immune privileged” and devoid of a functional lymphatic system (9). This view was overturned in 2015, when Louveau and Kipnis identified functional lymphatic vessels lining the dural sinuses while searching for T−cell gateways into and out of the meninges (8, 9). Subsequent work confirmed the presence of these vessels in humans and non-human primates through non-invasive MRI and histopathology (17, 18). MLVs were shown to connect with deep cervical lymph nodes (dCLNs) and to drain CSF as well as immune cells (8, 19). (**Figure 1**)

**Figure 2:**
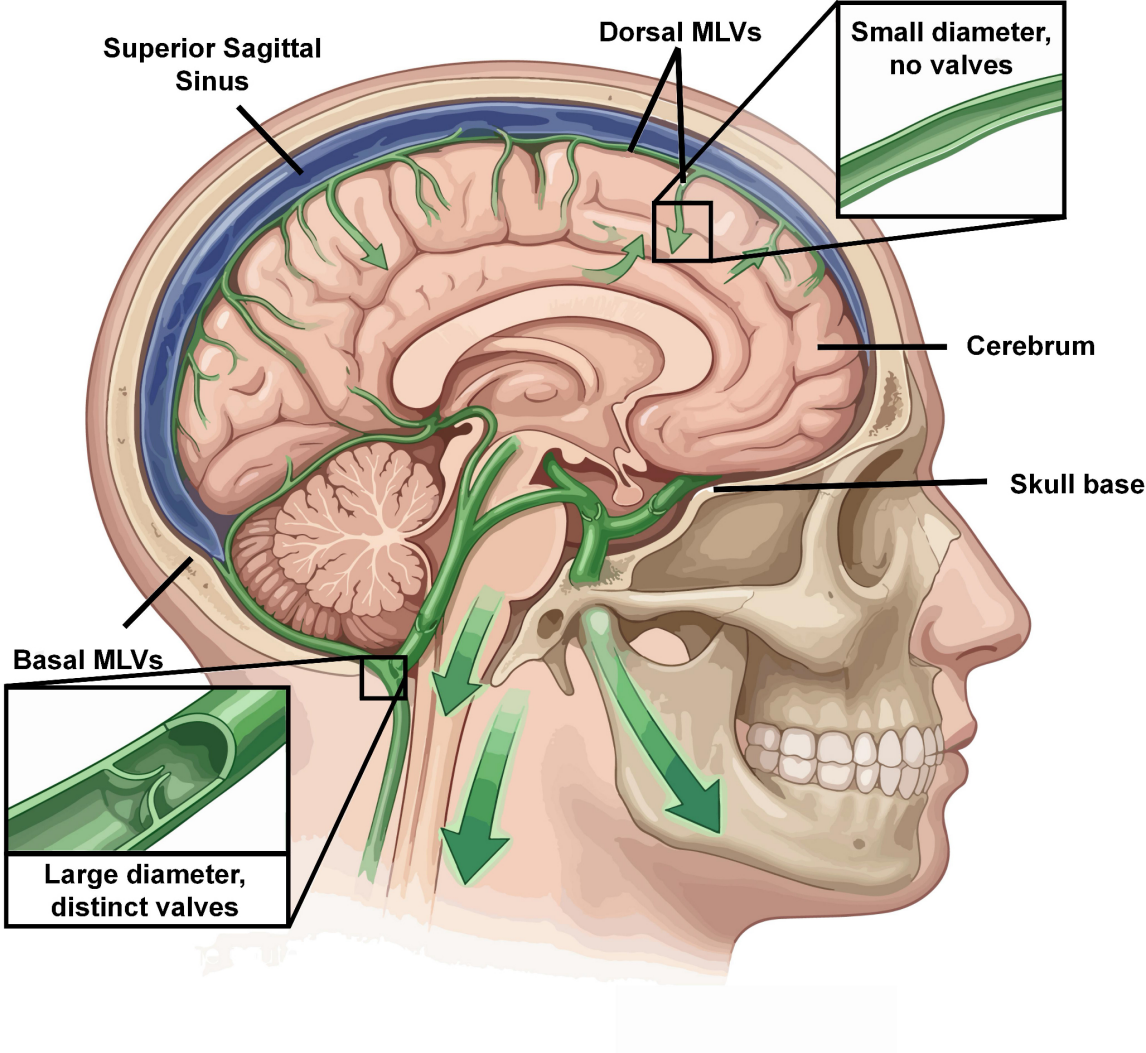
Anatomical distribution and structural characteristics of dorsal and basal meningeal lymphatic vessels (MLVs). A mid-sagittal view of the brain illustrating the distinct anatomical features of the meningeal lymphatic network. The dorsal MLVs (top), running adjacent to the superior sagittal sinus, are characterized by their small diameter and a lack of lymphatic valves. In contrast, the basal MLVs (bottom), located at the skull base, exhibit larger diameters and possess distinct intraluminal valves. These robust basal networks serve as the primary conduits for the drainage of cerebrospinal fluid (CSF) and brain-derived molecules toward the deep cervical lymph nodes.

MLVs reside mainly in the dura mater and are subclassified into dorsal and basal groups. Dorsal MLVs course along the superior sagittal and transverse sinuses; they have small diameters, lack valves, and possess incomplete walls, suggesting a modest contribution to CSF drainage under physiological conditions (8, 19). On the other hand, basal MLVs are located at the skull base, close to the olfactory bulb and cranial-nerve exits. Unlike the smaller, valve-less dorsal vessels, basal MLVs possess larger diameters, branching patterns, and functional valves, thereby constituting the principal anatomical pathways for CSF and immune-cell trafficking (8, 9). Similar dorsal-basal structural dichotomies and functional connections to dCLNs have also been established in humans and non-human primates. Such evolutionary conservation indicates a fundamental and indispensable role for MLVs in central immune regulation across mammalian species (11, 17, 20). (**Figure 2**)

### 2.2 Physiological and pathological functions in fluid homeostasis

MLVs constitute major anatomical routes for CSF and solute clearance from the cranial cavity (21). Basal MLVs serve as the principal routes for CSF drainage, with “button-like” endothelial junctions that allow macromolecular efflux (22). Approximately 50% of CSF exits the cranium via MLVs into the dCLNs (23), while the remainder is absorbed by arachnoid granulations (24, 25). Experimental ablation of basal MLVs elevates intracranial pressure, whereas induction of lymphangiogenesis, for example via VEGF-C, has been shown to enhance drainage and alleviate hydrocephalus (26). MLVs also cooperate with the glymphatic system to eliminate metabolic waste (11, 27). β−Amyloid is transported across the lymphatic endothelium via receptor−mediated mechanisms, and impaired drainage precipitates parenchymal Aβ deposition, thereby linking MLVs dysfunction to Alzheimer’s disease pathology (10, 20). Within the brain tumor niche, the meningeal lymphatic and glymphatic systems play a crucial role in the clearance of metabolic waste. However, tumor-induced mechanical compression impairs this specialized fluid drainage network, exacerbating the accumulation of metabolites such as lactate, which may contributes to local acidosis and peritumoral edema. Thereby fostering a hostile, immunosuppressive microenvironment that promotes malignant cell proliferation and therapeutic resistance (13, 28). Taken together, efficient meningeal lymphatic drainage is integral to intracranial fluid and metabolite homeostasis, and its disruption or remodeling can directly reshape the tumor-relevant microenvironment. In recent years, advanced magnetic resonance imaging (MRI) techniques have provided non-invasive approaches to visualize and assess MLV structure and function. Sequences such as black-blood MRI, which suppresses vascular flow signals to enhance contrast of the lymphatic wall, and diffusion tensor imaging (DTI), which tracks fluid diffusion pathways, have been applied to delineate meningeal lymphatic routes and evaluate their drainage efficiency. These imaging modalities offer valuable tools for studying dynamic changes of brain lymphatic system under both physiological and pathological conditions (17, 29).

In parallel, MLVs orchestrate immune surveillance by bridging central and peripheral immunity (30). Antigen-presenting cells such as dendritic cells and microglia capture pathogens or tumor antigens in the CNS and migrate along MLVs to the dCLNs where they initiate T-cell responses (13, 19). The integrity of dorsal MLVs is essential for efficient T-cell priming, and their ablation has been shown to diminish responses to immunotherapy in murine glioma models (13). In addition, MLVs display structural plasticity that tunes immune access. Under physiological conditions, their limited permeability restrains peripheral leukocyte entry. Under pathological conditions, tumor-derived VEGF-C drives MLV expansion and lumen dilation, which actively enhances CCL21/CCR7-dependent T-cell and antigen-presenting cell trafficking toward the deep cervical lymph nodes (12). In brain metastasis models, increased MLV drainage promoted intratumoral T-cell infiltration and improved survival (13). These findings demonstrate that MLVs actively regulate CNS immune surveillance, and changes in their structure can either support or compromise anti-tumor immunity.

## 3 Brain metastases: key features and challenges

### 3.1 Pathophysiology of brain metastasis

Brain metastasis is a multi-stage process that depends on dynamic interactions of disseminated tumor cells (DTCs) with the host microenvironment (2, 31). The metastatic cascade involves detachment from the primary tumor, intravasation, survival in the circulation, blood-brain barrier (BBB) penetration, and intracerebral colonization (32–35). Hematogenous dissemination is the predominant route (1). After breaching the vascular basement membrane, DTCs enter the systemic circulation, where shear stress and immune attack select for highly plastic sub-clones (36, 37). The brain’s rich vascularization favors entrapment of these cells in capillaries (38). To cross the BBB, tumor cells deploy diverse mechanisms: (i) secreting matrix metalloproteinases (MMP-2/9) to degrade endothelial tight-junction proteins (39), (ii) releasing miR-181c-loaded extracellular vesicles that downregulate BBB-stabilizing genes (40), or (iii) activating L1CAM/β1-integrin signaling to displace pericytes and open paracellular gaps (41). Once beyond the barrier, DTCs interact with astrocytes and microglia, which release supportive signals that promote colonization (42, 43). Crucially, the subsequent pathophysiology of established brain metastases involves profound remodeling of the local microenvironment. The disruption of the blood-tumor barrier leads to severe vasogenic edema and elevated interstitial fluid pressure (IFP). Concurrently, the expanding tumor recruits regulatory T cells (Tregs) and reprograms tumor-associated macrophages (TAMs) to create an immunosuppressive niche. These dynamic biomechanical and immunological changes in the brain parenchyma tightly interact with the CNS fluid drainage systems, ultimately driving the functional remodeling of the meningeal lymphatic network (5).

Furthermore, peripheral tumors draining to dCLNs might exploit the lymphatic network under pathological conditions. Although physiological lymphatic flow is strictly anterograde, severe lymphatic valve dysfunction and nodal blockage by tumor masses might alter local fluid dynamics. While direct tumor retrograde flow through MLVs requires further *in vivo* validation, recent nanoparticle delivery models have demonstrated the anatomical feasibility of retrograde transport from the cervical lymphatic network to the brain, suggesting a potential access route during advanced metastatic disease (44). In addition, breast and lung cancers can metastasize to the choroid plexus or leptomeninges through the bloodstream, from which tumor cells disseminate within the CSF to seed diffuse meningeal disease (45). Primary tumors of the skull base, such as nasopharyngeal carcinoma, may directly infiltrate the intracranial cavity via osteodural destruction, producing focal metastases (46).

### 3.2 Clinical limitations and therapeutic challenges of brain metastasis

Despite progress in surgery, radiotherapy, and targeted therapy, brain metastases remain associated with dismal outcomes, with median survival often measured in months (7). Several barriers underlie this poor prognosis. First, the BBB prevents the penetration of most systemic agents (47). Second, the intracranial milieu is strongly immunosuppressive, favoring immune evasion and blunting the effectiveness of systemic immunotherapy (48, 49). Third, marked inter-lesional and molecular heterogeneity contributes to therapeutic resistance. Lesions arising from different primaries, such as EGFR/ALK-mutated lung cancers or BRAF-mutated melanomas, display distinct molecular profiles and drug sensitivities (50–52). Even within a single patient, individual metastases differ in vascularity, immune infiltration, and metabolic adaptations, reflecting clonal evolution and local selection pressures (53–56). Such inter- and intra-tumoral heterogeneity complicates treatment and promotes recurrence (57). These pathophysiological and clinical challenges illustrate why brain metastases remain one of the most difficult complications of systemic cancer. Traditionally viewed as absent from the CNS, these vessels have now been recognized as routes for CSF drainage, antigen transport, and immune regulation (12). Their anatomical position and functional plasticity suggest they could influence both tumor dissemination and anti-tumor immunity. However, targeting MLVs presents a significant therapeutic paradox. While enhancing MLV drainage could boost anti-tumor immunity by increasing antigen presentation, it simultaneously risks facilitating the retrograde or anterograde dissemination of tumor cells. This dualistic nature creates a narrow therapeutic window and necessitates precise spatiotemporal control over MLV modulation, a challenge further elaborated in Section 4 and 5.

## 4 Dual roles of MLVs in brain metastases: friend and foe

**Figure 3:**
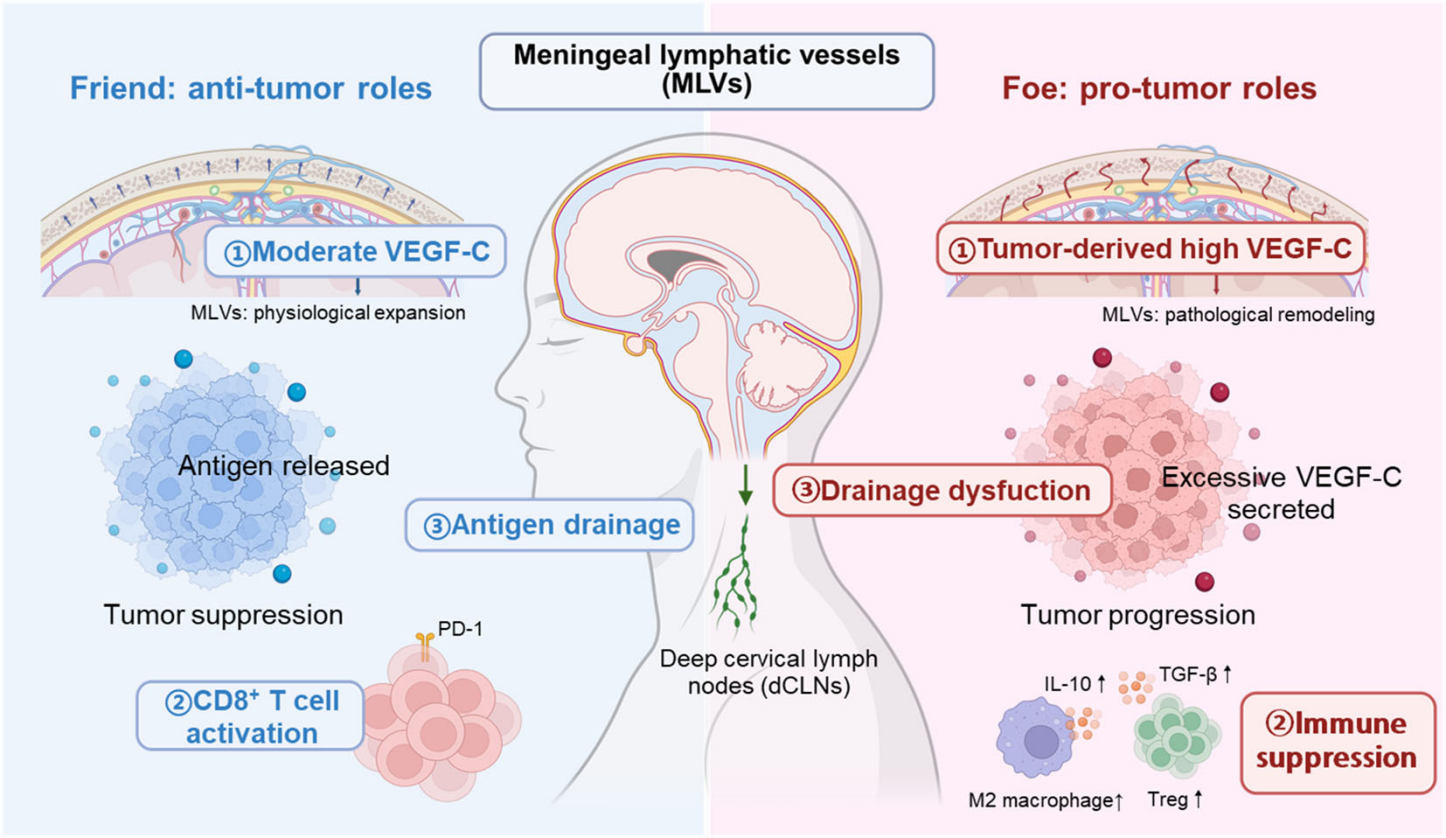
Dual roles of meningeal lymphatic vessels (MLVs) in brain tumor immunity. MLVs can exert both anti-tumor and pro-tumor functions depending on the context of VEGF-C signaling. On the left, moderate VEGF-C induces physiological MLV expansion, facilitating antigen drainage to deep cervical lymph nodes (dcLNs), enhancing CD8^+^ T cell activation, and promoting tumor suppression, partly through PD-1–mediated immune responses. On the right, excessive tumor-derived VEGF-C drives pathological MLV remodeling, leading to drainage dysfunction, immune suppression, and tumor progression. This is associated with increased TGF-β1, IL-10, M2 macrophages, and regulatory T cells (Tregs). The schematic highlights the paradoxical roles of MLVs as both “friend” and “foe” in brain tumor immunity.

As depicted in **Figure 3**, MLVs can function as either “friend” or “foe” depending on their structural integrity and the tumor microenvironment. MLVs are increasingly recognized as regulators of CNS immunity. In brain metastases, they act as a double-edged sword: under physiological conditions supporting anti-tumor responses, but under tumor-driven remodeling facilitating dissemination and immune evasion.

### 4.1 Friend: antigen drainage and immune activation

MLVs serve as conduits for CSF and antigen drainage into dCLNs, where antigens are processed by dendritic cells and presented to T lymphocytes (30, 58). This pathway links the CNS to peripheral immunity and enables systemic anti-tumor responses. Animal studies demonstrate that intact dorsal MLVs are indispensable for effective T-cell priming. For example, ablation of these vessels in glioma models markedly reduced the efficacy of immune checkpoint blockade (anti-PD-1/CTLA-4) by limiting antigen transport to lymph nodes and subsequent T-cell activation (12, 13).

In the context of brain metastases, enhancing lymphatic drainage has been shown to increase intratumoral CD8^+^ T-cell infiltration and prolong survival (12, 13). VEGF-C–mediated expansion of MLVs, when appropriately regulated, can amplify antigen flow, improve T-cell priming, and strengthen immunotherapy responses (12, 14). These findings suggest that functional MLVs may serve as allies in restoring effective anti-tumor immunity within the otherwise immunosuppressive intracranial milieu (20). Mechanistically, this immunological benefit is driven by the molecular signaling events downstream of vascular endothelial growth factor C (VEGF-C), which mediate the remodeling of the lymphatic vasculature to maintain both antigen trafficking and the viability of lymphatic endothelial cells. The binding of tumor-derived VEGF-C to VEGFR-3 triggers the phosphorylation of specific tyrosine residues, which subsequently activates the PI3K/Akt/mTOR and MAPK/ERK signaling cascades. These pathways are indispensable for the proliferation and survival of lymphatic endothelial cells (LECs). Specifically, Akt activation promotes LEC survival within the stressful tumor microenvironment, while the ERK pathway drives the formation of pathological, enlarged lymphatic tubes, a hallmark of MLV remodeling in brain metastases.

### 4.2 Foe: tumor-driven remodeling and dissemination

Conversely, tumor cells can subvert MLV function. In brain metastases, this phenomenon was recently documented: black-blood MRI sequence imaging revealed MLV remodeling in human patients with metastatic lung cancer (59), while corresponding murine models have further confirmed altered MLV structure and impaired drainage function (60).

While the exact molecular drivers of this remodeling in metastatic tumors are still being elucidated, insights from primary brain tumors offer a plausible mechanistic paradigm. In gliomas, tumor-derived VEGF-C has been explicitly identified as a major factor inducing pathological lymphangiogenesis, characterized by vessel dilation, chaotic branching, and increased permeability (12). Whether metastatic tumors rely on similar VEGF-C-dependent pathways to induce the morphological changes observed clinically (59, 60) remains a hypothesis that warrants further investigation. Furthermore, the functional plasticity of MLVs is broad, as they mediate the drainage of various biological entities, including neurotropic viruses (61), highlighting the complexity of their role in responding to different pathological challenges.

These structural alterations yield morphologically aberrant MLVs, which potentially compromise the efficiency of anterograde drainage of cells and macromolecules from the CNS to the dCLNs. While physiological flow is anterograde, altered fluid dynamics are hypothesized to facilitate retrograde transport from dCLNs to the meninges via lymphovenous connections. Although demonstrated as anatomically feasible in nanoparticle models (44, 62), this retrograde flow is currently viewed more as a potential route for targeted drug delivery rather than a primary mechanism for spontaneous metastatic seeding.

**Figure 4:**
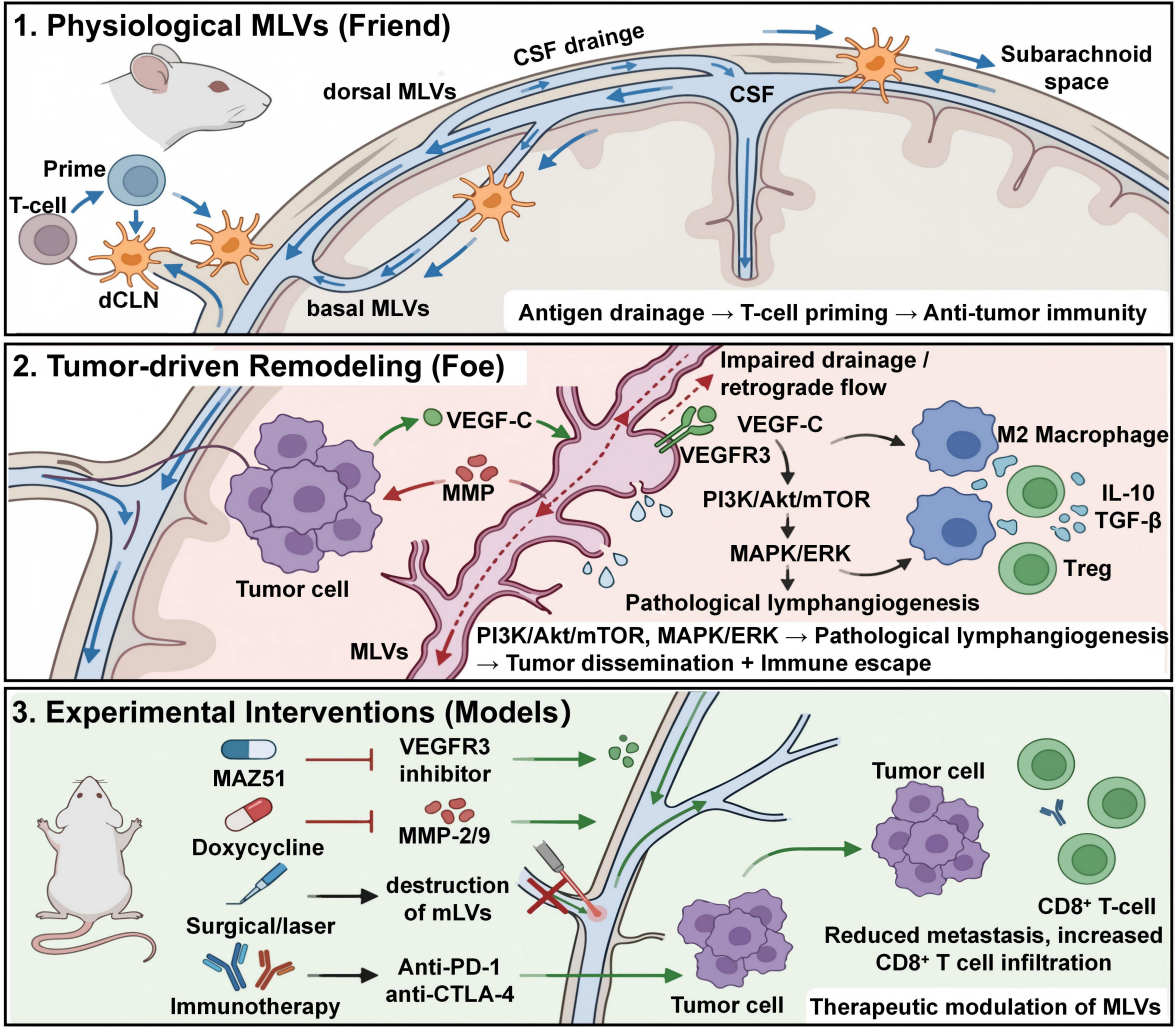
Mechanistic modeling of meningeal lymphatic vessel (MLV) remodeling and tumor dissemination in a murine model. Tumor cells inoculated into the cisterna magna secrete high levels of VEGF-C, which drives the pathological dilation of basal MLVs located at the skull base. This structural remodeling compromises lymphatic integrity and promotes lymphatic invasion by tumor cells. Consequently, these co-opted MLVs act as direct conduits that facilitate tumor cell drainage and subsequent metastatic seeding into the deep cervical lymph nodes (dcLNs). This *in vivo* model highlights the detrimental role of MLVs in exacerbating extracranial tumor dissemination.

MLV remodeling also contributes to intracranial hypertension and metabolic imbalance. Tumor-induced dilation can impair CSF drainage, while the growing tumor’s mass effect and edema compress dorsal MLVs, further slowing outflow (63). Functionally, this not only exacerbates neurological decline but also alters the tumor microenvironment by reducing waste clearance and favoring acidosis, conditions that promote malignant progression (13). (**Figure 4**)

### 4.3 Foe: immune suppression and escape

Perhaps more critically, remodeled MLVs facilitate immune evasion. Tumor-secreted cytokines drive microglia and macrophages toward an M2 phenotype, releasing IL-10 and TGF-β that suppress CD8^+^ T-cell cytotoxicity (48). Regulatory T cells (Tregs) accumulate within the intracranial niche, further dampening effector immunity and blocking dendritic cell maturation (49). Pathologically dilated and functionally impaired MLVs may preferentially channel immunosuppressive cells or soluble factors to deep cervical lymph nodes (dCLNs), while simultaneously failing to clear tolerogenic cytokines from the tumor microenvironment, thereby reinforcing both local and systemic immune escape (13).

Recent mechanistic studies highlight tumor-derived proteins such as HSP47, which induces microglial M2 polarization and suppresses T-cell activity (64)—an accumulation directly exacerbated by defective MLV clearance. In parallel, therapeutic interventions may paradoxically exacerbate immunosuppression. Tyrosine kinase inhibitors (TKIs), for instance, increase T-cell infiltration into brain metastases (65), but without functional MLVs to support continuous dendritic cell trafficking and fresh T-cell priming, these trapped infiltrates rapidly undergo exhaustion and upregulate CTLA-4 through NF-κB signaling, creating a state of “infiltration yet suppression” (12). Such findings illustrate how the tumor microenvironment and MLV remodeling converge to blunt otherwise effective immune responses.

Collectively, these data establish MLVs as paradoxical regulators of brain metastases. In their physiological state, they enable antigen drainage, promote T-cell priming, and enhance the efficacy of immunotherapy. Under tumor-driven remodeling, however, they may become permissive routes for tumor cell dissemination, sources of intracranial pressure dysregulation, and facilitators of immune suppression. Disentangling these opposing functions will be essential for developing strategies that harness the protective roles of MLVs while mitigating their tumor-promoting effects.

### 4.4 Heterogeneity across different primary tumors

The degree of MLV remodeling and VEGF-C expression exhibits significant variation based on the primary tumor origin. In non-small cell lung cancer (NSCLC) brain metastases, high levels of VEGF-C are frequently observed, which correlates with extensive peritumoral MLV dilation and early cervical lymph node involvement (66). In contrast, breast cancer brain metastases often exhibit a more diverse lymphangiogenic profile, where alternative factors such as VEGF-D or mechanical compression of the glymphatic-lymphatic interface may play a more prominent role (67). Whether VEGF-C concentrations in the CSF differ systematically between patients with NSCLC metastases and those with melanoma remains unclear, underscoring the need for tumor-type–specific evaluation before optimizing MLV-targeted therapies.

## 5 Therapeutic targeting of MLVs

Given their dual roles in brain metastases, MLVs have become attractive yet complex therapeutic targets. Approaches can be broadly divided into strategies that enhance their beneficial functions and strategies that restrict their tumor-promoting effects.

### 5.1 Enhancing meningeal lymphatic vessel function to improve immune response

Recent studies have revealed that increased flow velocity of MLVs has an important positive impact on the treatment of brain tumors. Wang et al. reported that patients with intracranial tumors display enhanced MLVs inflow but impaired outflow (68). VEGF-C restores MLV outflow, raising drug levels in brain metastases and improving prognosis (12, 13). Hu et al. showed that VEGF-C/CCL21 activation boosts MLV-mediated transport, enhancing radiotherapy and immunotherapy (13). Furthermore, combining previous work, physical interventions (light exposure (69), neck massage (70), or exercise (71)) have been shown to improve baseline meningeal lymphatic drainage, although these findings are currently established primarily in physiological and neurodegenerative models. Translating these non-invasive strategies to neuro-oncology represents an intriguing translational frontier. Theoretically, safely enhancing MLV clearance through these physical modalities could synergize with pharmacological measures, becoming potential adjunct methods to enhance the efficacy of brain metastasis therapies.

### 5.2 Inhibiting MLV remodeling to block tumor dissemination

While the specific clinical inhibition of MLVs in brain metastases is still an emerging field, robust evidence from extracranial highly metastatic tumors, such as peripheral melanoma and lung cancer, provides strong proof-of-concept that inhibition of lymphatic remodeling can reduce tumor dissemination. For example, in peripheral tumor models, Lee and colleagues reported that the selective VEGFR3 inhibitor MAZ51 blocks the VEGF-C/VEGFR3 axis. This blockade markedly suppresses melanoma cell-induced lymphangiogenesis and decreases the incidence of lymph-node metastasis (72). Additionally, García-Caballero and colleagues demonstrated that doxycycline inhibits tumor-secreted MMP-2 and MMP-9. Consequently, extracellular-matrix degradation is reduced, lymphatic dilation is limited, and tumor cell dissemination along lymphatic routes is attenuated (73). These findings provide experimental support for combined anti-angiogenic and anti-lymphangiogenic strategies across multiple solid-tumor models.

Although these foundational studies focused on peripheral lymphangiogenesis, they establish a critical pharmacological rationale for neuro-oncology. Given that brain tumors and cerebral metastases similarly exploit the VEGF-C/VEGFR3 axis and secrete MMPs to drive pathological MLV expansion (12), repurposing these anti-lymphangiogenic agents could theoretically block MLV-mediated tumor escape to the dCLNs. Thus, these findings from peripheral solid tumors provide a strong rationale for investigating combined anti-angiogenic and anti-lymphangiogenic therapies in the setting of advanced brain metastases.

### 5.3 MLV-targeted drug delivery systems

Recent evidence indicates that modulation of the retrograde MLVs route can markedly improve immunotherapy for breast-cancer brain metastases.

Dai and colleagues developed a phospholipid nanosystem (α-PLNP). After subcutaneous injection into the neck, the particles travel retrogradely along MLVs into the brain (62). Melittin-loaded α-M-PLNPs activate antigen-presenting cells within deep cervical lymph nodes and promote CD8^+^ T-cell infiltration into metastatic lesions, thereby extending survival in murine models. Zhao and colleagues reported that indocyanine-green-loaded PLGA nanoparticles delivered via the same route increase brain drug exposure 44-fold compared with intravenous administration (15). The formulation achieved effective photodynamic therapy in a glioblastoma model. Moreover, Ramos-Zaldivar and colleagues demonstrated that specifically engineered/optimized nanoparticles exhibit an 8.8% brain-targeting efficiency after cervical injection, utilizing lymphatic routes to significantly exceed the CNS accumulation of non-targeted or systemic controls (44). Collectively, these studies validate the peripheral-MLV retrograde pathway as an efficient and minimally invasive route for delivering chemotherapeutics or immunomodulators while markedly reducing systemic toxicity.

## 6 Challenges and prospects

Despite the therapeutic potential of MLV-targeted approaches for brain metastases, significant hurdles must be overcome, with progress relying on interdisciplinary advances in basic science and clinical application.

### 6.1 Challenges in anatomical and functional heterogeneity

Human MLVs exhibit marked inter-individual heterogeneity in both distribution density and functional capacity. Post-mortem and non-invasive high-resolution imaging analyses reveal substantial anatomical variation in basal skull MLV density among adults (72). Furthermore, clinical and preclinical studies establish that age-driven endothelial senescence precipitates structural deterioration and a substantial loss of MLV density (10).

Such heterogeneity directly influences the response to VEGF-C-mediated enhancement. After VEGF-C treatment, aged murine models show only a 1.3-fold increase in lymphatic drainage, whereas young mice achieve a 2.8-fold increase. Regional differences further complicate outcomes. Therefore, both individual and regional factors should be considered in clinical applications.

### 6.2 Balancing dual roles of MLVs

Navigating the “double-edged sword” of MLV modulation is a major clinical dilemma. Stimulating MLVs improves drug delivery and immune infiltration, but widened lymphatic conduits concurrently risk accelerating tumor dissemination. As seen in GBM models, VEGF-C-driven lymphangiogenesis boosts CD8^+^T-cell recruitment but simultaneously enables metastatic escape (12). Additionally, therapeutic windows are highly tumor-specific; early colonization phases may offer peak responsiveness for certain metastases, while others require sustained intervention due to chaotic lymphatic remodeling (74). Therefore, precision medicine approaches will be essential to harness MLVs as a “friend” while mitigating their potential as a “foe.”

### 6.3 Barriers to clinical translation

Significant differences between preclinical models and human pathology impede the clinical translation of research findings. Species-specific variations result in anatomical discrepancies between humans and mice; therefore, data from animal experiments cannot be directly extrapolated to humans. Magnetic resonance imaging has been adopted to detect MLVs, yet technical challenges persist. Although recent advances, such as black-blood MRI and diffusion-based sequences, have improved visualization to some extent, their sensitivity and standardization remain limited, especially for assessing dynamic lymphatic function *in vivo*. In critical regions of the skull base, the resolution is still insufficient for precise quantification. Moreover, longitudinal studies that characterize dynamic changes in MLV function during the progression of brain metastases are currently lacking. This absence constrains the development of stage-specific therapeutic strategies.

### 6.4 Future directions

Future research must focus on three key areas to advance MLV-targeted therapies for brain metastases: 1. Developing non-invasive imaging techniques to characterize MLV structure and function in human patients. High-resolution contrast-enhanced MRI with lymphotropic tracers has shown promise in preclinical models, enabling quantification of MLV characteristics and potentially allowing longitudinal monitoring of MLV remodeling during treatment. 2. Currently, no completed clinical trials have specifically investigated MLVs in patients with brain metastases; however, most evidence remains preclinical, underscoring the need for rigorously designed early-phase trials. Future research should focus on engineering bifunctional drug delivery systems that integrate lymphatic targeting with immunomodulation. 3. Overcome barriers to clinical translation and reduce the gap between preclinical models and human pathology.

In summary, MLVs offer a critical framework for understanding disease progression and therapeutic development. With advancing research, MLVs-targeted strategies hold significant potential to transform the prognosis of patients with brain metastases, bridging the gap between preclinical innovation and clinical benefit.
